# Reverse translation of artificial intelligence in glaucoma: Connecting basic science with clinical applications

**DOI:** 10.3389/fopht.2022.1057896

**Published:** 2023-01-04

**Authors:** Da Ma, Louis R. Pasquale, Michaël J. A. Girard, Christopher K. S. Leung, Yali Jia, Marinko V. Sarunic, Rebecca M. Sappington, Kevin C. Chan

**Affiliations:** 1 School of Medicine, Wake Forest University, Winston-Salem, NC, United States; 2 Atrium Health Wake Forest Baptist Medical Center, Winston-Salem, NC, United States; 3 School of Engineering Science, Simon Fraser University, Burnaby, BC, Canada; 4 Department of Ophthalmology, Icahn School of Medicine at Mount Sinai, New York, NY, United States; 5 Ophthalmic Engineering & Innovation Laboratory (OEIL), Singapore Eye Research Institute, Singapore National Eye Centre, Singapore, Singapore; 6 Duke-NUS Medical School, Singapore, Singapore; 7 Institute for Molecular and Clinical Ophthalmology, Basel, Switzerland; 8 Department of Ophthalmology, The University of Hong Kong, Hong Kong, Hong Kong SAR, China; 9 Casey Eye Institute, Oregon Health & Science University, Portland, OR, United States; 10 Institute of Ophthalmology, University College London, London, United Kingdom; 11 Departments of Ophthalmology and Radiology, Neuroscience Institute, NYU Grossman School of Medicine, NYU Langone Health, New York University, New York, NY, United States; 12 Department of Biomedical Engineering, Tandon School of Engineering, New York University, New York, NY, United States

**Keywords:** deep learning, artificial intelligence, reverse translation, transfer learning, glaucoma, optical coherence tomography, visual field

## Abstract

Artificial intelligence (AI) has been approved for biomedical research in diverse areas from bedside clinical studies to benchtop basic scientific research. For ophthalmic research, in particular glaucoma, AI applications are rapidly growing for potential clinical translation given the vast data available and the introduction of federated learning. Conversely, AI for basic science remains limited despite its useful power in providing mechanistic insight. In this perspective, we discuss recent progress, opportunities, and challenges in the application of AI in glaucoma for scientific discoveries. Specifically, we focus on the research paradigm of reverse translation, in which clinical data are first used for patient-centered hypothesis generation followed by transitioning into basic science studies for hypothesis validation. We elaborate on several distinctive areas of research opportunities for reverse translation of AI in glaucoma including disease risk and progression prediction, pathology characterization, and sub-phenotype identification. We conclude with current challenges and future opportunities for AI research in basic science for glaucoma such as inter-species diversity, AI model generalizability and explainability, as well as AI applications using advanced ocular imaging and genomic data.

## 1 Introduction

### 1.1 Reverse translation in glaucoma studies

In therapeutic development research, the conventional forward translation paradigm adopts a benchtop-to-bedside scheme, which aims to find genomic associations or therapeutic biomarkers starting from *in vitro* studies and animal models to validation in human subjects for further refinements and therapeutic development. On the other hand, reverse translation offers a different research paradigm that starts from human studies to identify and generate hypotheses for validation in animal or *in vitro* studies (1). This alternative research paradigm is poised to address several bottlenecks in conventional forward translation due to its more patient-centered, seamless, continuous, and cyclical process (2).

In the field of glaucoma research, there is an urgent need to develop therapeutic methods beyond the conventional clinically proven therapeutic intervention of lowering intraocular pressure (IOP). Glaucoma is the leading cause of irreversible blindness worldwide. Although glaucoma is characterized by progressive damage of the retinal ganglion cells and their axons, very little is known about its underlying mechanisms. IOP is a major risk factor, but not the cause of the disease. However, only a limited number of current clinical trials (<7%) were able to focus on novel neurotherapeutic targets (3). Such a mismatch between the need and reality of the sub-phenotyping therapeutic development research is in part hindered by the limitations embedded in the conventional forward translation paradigm. Reverse translation could potentially alleviate these barriers. For example, recent work using a transgenic mouse model based on the optineurin E50K gene mutation, originally discovered in human normal-tension glaucoma patients (4), discovered novel mutation-level-dependent age effects on visual impairment (5). In another example, inspired by the relationship between aging and glaucoma in clinical settings, Lu et al. designed retinal tissue reprogramming through the induction of ectopic expression of the four Yamanaka transcription factors and showed reverse age-related vision loss and eye damage in an aging mouse model with glaucoma (6). These and other related studies demonstrated the successful implication of reverse translation in glaucoma research (7–9).

### 1.2 Role of AI in reverse translation

Universally, machine learning and artificial intelligence (AI) in medicine have been applied mostly in clinical data with far fewer studies pertaining to animal models. This disparity in application arises largely from greater availability and standardization of bedside clinical data compared to benchtop data. This is particularly true in the field of glaucoma, in which much of the recent technology development has been established in bedside clinical settings (10, 11). Disparities in technological advancement that favor clinical applications present an ideal opportunity to apply the reverse translation research paradigm. Thus, AI applications in glaucoma are poised for technical adaptations that can pioneer reverse translation from human to animal models. In this perspective, we summarize several areas of research opportunities for reverse AI translation in glaucoma. We also point out current challenges in the field, and identify several research directions to achieve successful reverse translation for scientific discoveries in glaucoma in the future.

## 2 Areas of research opportunities for reverse translation of AI in glaucoma

This perspective is structured in the following order: We first review the latest research using supervised classification AI models to predict glaucoma-related clinical and pathological conditions, such as rapid glaucoma progression (Section 2.1) and optic nerve head (ONH) abnormality detection (Section 2.2). Parallelly, we summarize the applications of unsupervised clustering AI algorithms to identify glaucoma subtypes (Section 2.3), such as novel archetypal visual field loss patterns and ONH-abnormality structural patterns. The next sections focus on AI algorithms to identify glaucoma-related risk factors (Section 2.4) and endophenotypes (Section 2.5–2.7). Specifically, we take a close look at AI-derived phenotypic biomarkers for glaucoma using ophthalmic imaging techniques including structural optical coherence tomography (OCT) (Section 2.5–2.6) and vascular OCT-Angiography (OCTA) (Section 2.7). Finally, we review recent glaucoma AI research that addressed some of the common AI challenges including AI model generalizability (Section 2.8), model explainability (Section 2.9), and model transferability through federated learning techniques (Section 2.10) to train aggregated models across multiple sites without the need of sharing data among participating sites. We conclude by discussing current opportunities and challenges for reverse AI translation in glaucoma (Section 3.1), and share our perspective on future research directions (Section 3.2) incorporating state-of-the-art explainable AI method development into cutting-edge ophthalmic imaging and genomic techniques.

### 2.1 Predicting the risk of rapid glaucoma progression

Predicting the disease progression or risk is important for patient stratification and guiding early intervention. Visual field measurement is a low-cost diagnostic tool for evaluating visual function. By using a deep neural network trained on low dimensional, baseline 2D visual field measurements, recent studies showed promising predictive power in forecasting the risk of rapid glaucomatous progression (12, 13). On the other hand, OCT may be capable of predicting visual field progression (14). The important question for future studies of reverse translation would be: which approaches would be more appropriate? Would visual field measurements predicting glaucoma risk have more promising values for clinical applications such as early screening or clinical trial participant stratification, while using OCT to predict visual fields fits better in animal studies to understand the structural-functional relationships within the pathogenic mechanisms of glaucoma? To answer these questions, longitudinal experiments could be designed in transgenic animal models to simultaneously evaluate the progression of functional and structural abnormalities and model their pathogenic cascades as a function of time.

### 2.2 Optic disc and optic nerve head abnormality detection in glaucoma

The ONH represents the confluence structure for the entire visual system. Glaucoma is associated with elevated IOP, whereas the ONH is heavily affected by the biomechanical forces due to elevated IOP and is therefore susceptible to structural damage and the associated functional loss. Han et al. trained a convolutional neural network (CNN) model on a large dataset of 282,100 images from both the UK Biobank and the Canadian Longitudinal Study on Aging - CLSA for automatic AI labeling of the ONH (15). Their study was able to extract two key ONH parameters: vertical cup-to-disc ratio and vertical disc diameter. Using OCT, Heisler et al. demonstrated auto-peripapillary region extraction in a clinical cohort with a much smaller dataset using a composited approach of peripapillary layer segmentation and Faster R-CNN-based object detection of Bruch’s membrane opening (BMO) (16). Accurate segmentation of the optic disc region and peripapillary retinal boundaries has also been demonstrated by combining CNN and multi-weights graph search (17). These results showed promising potentials for reverse translation to animal glaucoma models in which the sample size is much smaller compared to large-scale population studies such as the UK Biobank. It is worth noting that special care should be taken in the experimental design that, such reverse translation might mainly be applicable in animals with ONH anatomy similar to human eyes.

### 2.3 AI-derived data-driven glaucoma sub-phenotyping

Understanding disease subtypes is important to achieve precision medicine. For example, different subtypes of primary open-angle glaucoma (POAG) showed different patterns of visual field progression (18). Distinctive glaucoma sub-phenotypes were discovered based on visual field read patterns (19) or structural descriptions of the ONH shape models (20) through data-driven clustering and feature reduction methods such as Uniform Manifold Approximation and Projection (UMAP) and non-negative matrix factorization (21). Such clinically informed POAG sub-phenotypes provide opportunities for new frontiers of reverse translation. Existing AI studies in animal models are mainly limited to discriminating between glaucomatous and healthy eyes *via* OCT (22). With the reverse translation of AI findings from clinical studies, novel animal models can be developed or identified to understand the distinctive disease mechanisms for each of the POAG subtypes.

### 2.4 Genotype-associated genomic risk for glaucoma

**FIGURE 1:**
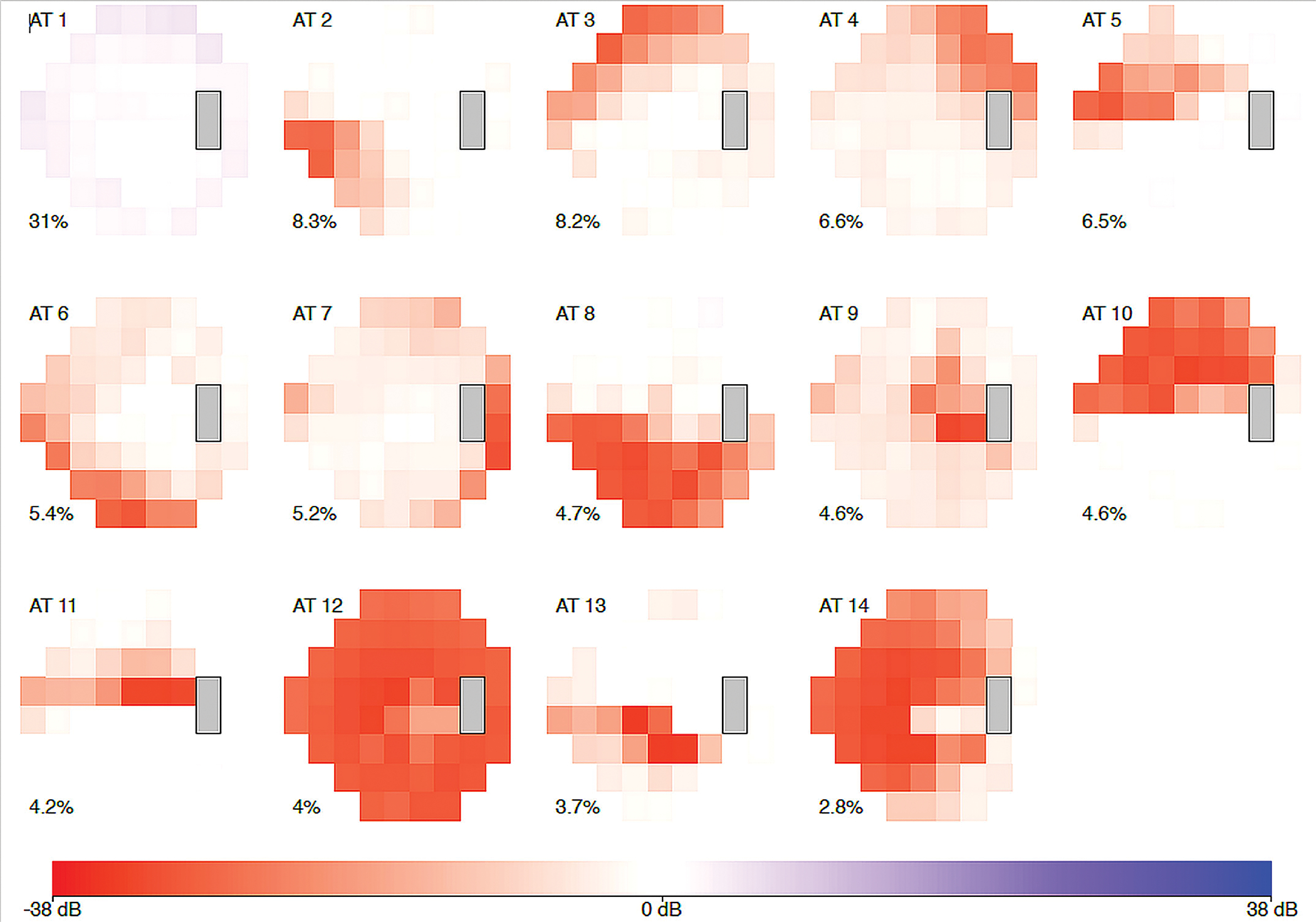
The 14 archetypal visual field loss patterns derived from visual fields of the 1957 incident primary open-angle glaucoma cases (2581 affected eyes). The integer at the top left of each archetype (AT) denotes the archetype number. The percentage at the bottom left of each archetype indicates this pattern’s respective average decomposition weight. The algorithm identified 14 archetypes: four representing advanced loss patterns, nine of early loss, and one of no visual field loss. African-American patients made up 1.3 percent of the study but had a nearly twofold increased risk of early visual field loss archetypes, and a sixfold higher risk for advanced field loss archetypes, when compared to white patients. [excerpted from (19)].

Genotype-phenotype associations are an important inter-species bridge to connect benchtop and bedside studies. A recent study has identified 14 archetypes of POAG using data-driven clustering methods based on the visual field measurement patterns (19) (Figure 1). By connecting the discovered sub-phenotypes with the ancestry data, the authors discovered the African-descendant ethnicity as the risk factor for specific POAG sub-phenotypes for both early and advanced loss archetypes. In another study, a genome-wide meta-analysis identified 127 open-angle glaucoma loci (23). AI-driven algorithms can also be used for assigning vertical cup-to-disc ratios to extend our knowledge about the genetic architecture of glaucoma (15). Such findings may guide the development of novel transgenic mouse models that are highly relevant to the human disease process. However, given the difference in ocular anatomy between the mouse and humans, it would be important to determine the inter-species translatability of the glaucoma sub-phenotypes, and animal-specific glaucoma sub-phenotyping models would need to be carefully trained and interpreted.

### 2.5 Morphological and biomechanical phenotype of the glaucomatous ONH

Describing the morphological and biomechanical phenotype of the ONH is critical for the field of glaucoma. Changes in ONH structure have been considered a central event in glaucoma, and the fragile ONH is constantly exposed to 3 major loads: IOP, cerebrospinal fluid pressure (CSFP), and optic nerve traction during eye movements. To better describe ONH structure in patients, Devalla et al. has proposed several deep learning approaches (e.g. DRUNET and ONH-Net) to simultaneously segment both connective and neural tissues of the ONH from OCT images (24), one of which was device-independent (25). Panda et al. and Braeu et al. employed these AI-driven approaches to identify novel morphological biomarkers for glaucoma in humans (20, 26). These technologies were then successfully ‘reverse-translated’ in the tree shrew model (27) to better understand non-linear optical distortions present in OCT images. This knowledge could ultimately improve our glaucoma predictions in patients.

Several technologies have been developed in humans to assess the biomechanics of the ONH. As it is now possible to observe IOP-induced (or gaze-induced) ONH deformations with OCT, techniques such as digital volume correlation, the virtual fields method, and other AI-driven approaches have been used to map local ONH tissue strain, biomechanical properties, and robustness (28–32). In a large glaucoma population, Chuangsuwanich et al. identified key biomechanical trends: (1) IOP-induced deformations were associated with visual field loss in high-tension glaucoma but not normal-tension glaucoma (33); and (2) normal-tension glaucoma ONHs were more biomechanically sensitive to changes in gaze, while high-tension glaucoma ONHs appeared more sensitive to changes in IOP (34). Similar techniques have in turn been used to test biomechanical hypotheses in non-human primates, allowing for a greater degree of freedom, and simultaneous control of IOP, CSFP, and blood pressure (35, 36). The knowledge gathered in those animal tests could ultimately help us refine a viable clinical test to assess ONH biomechanics in patients.

### 2.6 Retinal morphology and shape analyses for glaucoma

Biological and clinical explainability is important for both forward and reverse translations. Lee et al. developed a computational morphometric analysis pipeline to measure the individualized glaucoma-induced retinal structural changes through the estimation of retinal layer thicknesses and shape deformation over time (37). Such measurements require registration-based computation using a longitudinal dataset (38). Recent work by Shaini et al. used non-negative matrix factorization, an unsupervised dimensionality reduction and clustering method, to derive distinctive subphenotypes of ONH and peripapillary retinal nerve fiber layer (RNFL) surface-shape-based features, which could further improve the prediction accuracy of subsequent glaucomatous visual field loss (21).

**FIGURE 2:**
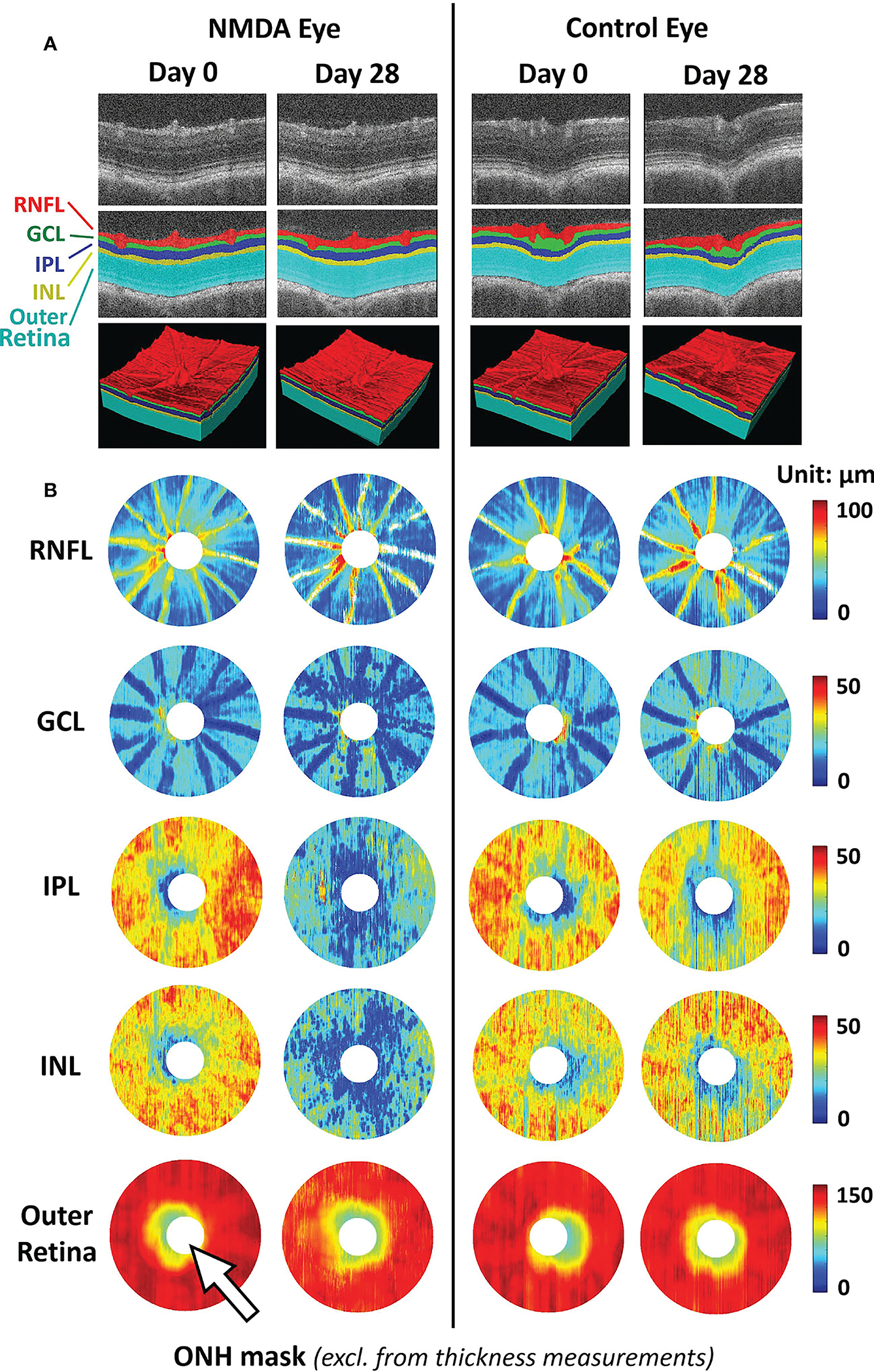
Representative images of deep learning-assisted automatic retinal layer segmentation **(A)** and the thickness measurements of 5 retinal layers for both injured and control rat eyes **(B)** before and 28 days after unilateral N-methyl-D-aspartate (NMDA) injection. Automatic retinal layer segmentation was achieved using LF-UNet - an anatomical-aware cascaded deep-learning-based retinal optical coherence tomography (OCT) segmentation framework that has been validated on human retinal OCT data (42). In this work, two techniques were applied to improve the efficiency and generalizability of the LF-UNet segmentation framework when training with a small, labeled dataset – 1) composited transfer-learning and domain adaptation, and 2) pseudo-labeling. [excerpted from (42)]. (RNFL, retinal nerve fiber layer; GCL, ganglion cell layer; IPL, inner plexiform layer; INL, inner nuclear layer; ONH, optic nerve head).

Focusing on the RNFL bundles, Leung et al. developed an optical texture analysis (39), and illustrated its diagnostic assessments for both glaucoma and non-glaucoma optic neuropathies (40). Such mathematical-driven modeling tools can be applied to both clinical studies and animal models to understand the relationships between RNFL integrity and other structural pathologies such as retinal vascular disruption. Furthermore, the model can further benefit from more accurate RNFL segmentation using deep-learning-based segmentation. Deep learning-based methods enable the automatic segmentation of retinal layers with high accuracy (41). Compared to clinical OCT, animal studies have a limited sample size and labeled ground truth data for training. In this sense, transfer learning and pseudo-labeling are proven to be beneficial in utilizing deep-learning models pre-trained on larger-scale clinical OCT data in animal studies. They require limited training data and minimal ground truth labels (42) (Figure 2).

Recent work by Brau et al. incorporated state-of-the-art geometric deep learning to train classifiers using point-cloud data derived from segmented ONH boundaries (26, 43). By using a dynamic-graph convolutional neural network (DGCNN), an explainable AI method, the authors were able to identify the critical 3D structural features of the ONH that are important to provide an improved glaucoma diagnosis. Some of those regions showed a great level of colocalization to the central retinal vessels, which aligns with their findings that the central retinal vessel trunk and branches have stronger diagnostic power for glaucoma compared to RNFL thickness (44). This model is also currently under clinical assessment (45). Importantly, this model can become a useful area of analysis with recent AI developments in glaucoma animal models. In OCT images, Choy et al. employed AI technology to delineate Schlemm’s canal lumens in mouse eyes (46). Similar segmentation and shape analysis approaches were recently applied to fixed tissues for automated analysis of multiple retinal morphological changes, including RNFL thickness (42), optic nerve density, and retinal ganglion cell soma density in animal models (47, 48). These studies suggest that, while population sizes for ground truth training may impose an obstacle to reverse translation of AI technology, they are not insurmountable.

### 2.7 AI in OCT angiography for glaucoma

OCT Angiography (OCTA) is a functional extension of OCT, which allows the detection of vascular-related retinal and optic nerve diseases (49, 50). OCTA has been used to quantify ONH blood flow in glaucoma since 2012 (51). Clinical studies show that OCTA-based vessel density and flow index are lower in glaucomatous eyes in the regions of the optic disc (52), peripapillary retina (53), and macular retina (54). OCTA-based vessel density can also reflect the severity of visual field loss in glaucoma patients (55). Recently, OCTA-derived nerve fiber layer plexus measurements have been used to further correlate with visual functions by simulating sector-wise visual field (56). Now, it is possible to detect glaucomatous focal perfusion loss using OCTA (57). Given the close relationship between the glaucoma-related pathologies and the retinal vascular regions (26), validated by other imaging modalities (44), we believe OCTA may serve as a new tool for glaucoma diagnosis and monitoring, and also understanding the mechanism of disease development (58, 59).

However, OCTA data may suffer from projection artifacts and motion artifacts (60). AI methods (61) have been applied for projection artifact removal (60, 62). AI has also been applied to enhance the retinal capillaries (63, 64), segment retinal vessels (65, 66), quantify the avascular zone (67–71), and map arteries and veins (72) in OCTA. There are potential opportunities for side-by-side development in humans with the intent to reverse translate.

### 2.8 AI model generalizability

Model generalization across devices is a crucial but challenging issue in AI development. Devalla et al. proposed a “3D digital staining” approach (25) that uses an “enhancer” neural network to learn the mathematical morphological operations to enhance the raw OCT images, which enables the application of a pre-trained segmentation of the ONH on different devices, thereby reducing variability. Similar approaches can be achieved through domain-adaptation using the generative adversarial network (GAN), with the potential to add additional shape or feature priors and constraints into pre-trained segmentation models (41) such as the source domain, structural similarity, signal-to-noise ratio, and high-level perceptual feature (73). Such data harmonization approaches can be important when translating to animal studies to account for different experimental setups and eliminate potential batch effects.

### 2.9 Explainable AI for glaucoma

The explainability of AI modeling is crucial for translational applications in both clinical and pre-clinical animal studies. Recent studies have shown that explainable visualization can identify previously non-reported regions surrounding ONH that may be associated with glaucoma pathogenesis (74, 75). Some of the conventional explainable AI methods, such as Grad-CAM, are limited in terms of showing localized feature-important saliency maps due to the lack of resolution. With the integration of B-scan aggregation and enface projection for AI model visualization method (76), it is feasible to identify localized pathological signatures that differentiate retinal disease subtypes with a relatively small training set using feature agnostic AI classifier without the need for labeling of pathological regions. More excitingly, the newly proposed biomarker activation map (BAM) is an explainable visualization method specifically designed for AI-based disease diagnosis (77). The generated BAMs were designed to only localize the AI model-utilized unique biomarkers belonging to the positive class and showed much higher localization capability (77) than Grad-CAM or other conventional explainable AI methods, such as attention maps. It is foreseeable that such techniques can also be used to identify glaucoma pathogenesis and validate biomarkers in future preclinical studies.

### 2.10 Federated learning for glaucoma

The development of robust and generalizable deep learning models usually requires large samples of representative training data, which might demand the aggregation of data from different location sources. However, data sharing for clinical data is often restricted due to patient privacy concerns. Federated learning, or collaborative learning, helps to resolve such barriers through the training of an aggregated model without the need for data transfer, and is important for accelerating AI glaucoma research. The work by Lo et al. demonstrated the successful implementation of the federated framework to train more generalizable aggregated models of retinal vessel segmentation and diabetic retinopathy classification using OCT data from three different institutions and different OCT machines with distinctive distributions of disease severity (78). With regard to glaucoma, the work by Christopher et al. exemplified the successful training of federated models for glaucoma detection using data from two institutions containing distinctive racial populations (79). For accelerating reverse translation into basic science, future studies are envisioned that involve collaborative efforts and openness in building shared data sources of preclinical imaging, biochemical, and behavioral modalities in healthy animals across species, age, and gender, as well as experimental high-tension and normal-tension glaucoma disease models for establishing robust baselines and predicting neurobehavioral changes for further research. For pre-clinical animal data, the barriers to data sharing would be lower without privacy concerns. Moreover, the techniques developed in the federated learning framework, such as parallel model training with normalized weighted sharing and dataset-specific domain adaptation, would be beneficial for reverse translation.

## 3 Discussion

### 3.1 Opportunities and challenges for reverse translation of AI in glaucoma

In the recent decade, extensive efforts have been put into the development and investigations of AI methods for glaucoma research in clinical settings. Not only did these studies show promising results in improving clinical outcomes on the bedside, they have also provided precious first-hand experiences that researchers can learn from towards reverse translation to animal studies. The data-driven findings of glaucoma-related genomic loci and glaucoma sub-phenotypes provide information for potential novel transgenic animal models to further study the biological mechanisms for precision medicine. The effectiveness of using OCT to predict visual field progression, as well as using visual field measurements to predict the risk of accelerated glaucoma progression, indicates strong functional-structural correlations towards the disease progression. Such insights will likely guide the experimental design of future animal studies to focus on specific pathophysiological and functional pathways of the disease mechanisms. Furthermore, many imaging-based AI models are readily translatable to animal-based pre-clinical studies, from structural segmentation to shape and biomechanical models for the ONH and optic disc, peripapillary retinal vascular pathology and avascular zone abnormality detection, as well as retinal nerve fiber bundle texture analysis. Finally, AI methods for model generalizability, domain adaptation, and explainable AI are crucial for reverse translation to evaluate the applicability of animal models to monitoring glaucomatous conditions.

To date, most of the AI method developments in glaucoma are focused on clinical applications, leaving much room for reverse translation to benchside basic science research. Some of the current challenges in reverse translation research include the anatomical differences in the visual system between human and animal models. For example, human and rodent eyes have different sizes, with fovea and lamina cribrosa being present in humans but not in rodents (though they possess the pseudofovea and glial lamina), and with optic nerve fibers decussating at the optic chiasm to the contralateral hemisphere to different extents between humans (52%) and rodents (above 90%). Finally, like other biomedical and clinical applications, AI applications in glaucoma research also inherit some of the current limitations, such as model interpretability and generalizability.

### 3.2 Future research directions

Future research on reverse translation for glaucoma can further benefit from integrating state-of-the-art developments in AI methods. Advanced AI models such as vision transformers have shown better generalizability in tasks depicting POAG when applied to diverse independent datasets (80). The fast-growing self-supervised learning techniques have shown promising applications for efficiently utilizing relatively large amounts of unlabeled data to learn pathological features that are not specific to certain diseases but generalizable to other diagnoses, such as patients with both glaucoma and diabetic retinopathy or age-related macular degeneration. Although this approach can be more practical as comorbidities often occur in patients, care should be taken when applying self-supervised learning in medical image data in which pathology-related variations are highly localized. This often causes the algorithm subject to shortcut learning, detecting non-clinically-relevant easy features to drive prediction (81). Therefore, it is essential to incorporate domain-specific information when developing self-supervised learning methods to avoid contamination by spurious features (82). This can also improve robustness when training on clinical problems with small sample sizes by bootstrapping the performance to achieve diagnosis-level explanation (83). Future research on explainable AI should not only resolve where the model focuses, but also how the changes in those locations affect the model performance. The counterfactual approach (84) could potentially help to unveil the blackbox of the deep learning models by interrogating the explainability of the internal layers of the neural network, leading to the causal explanation inside the model (85, 86).

Successful efforts in the reverse translation of AI may also benefit the development and use of large animal models of glaucoma (87–90). While larger animals, such as dogs, swine, and primates, have greater anatomical homology to humans, their use is limited by the cost of cohorts and ethical considerations. Tree shrews may be considered as an alternative glaucoma animal model given the presence of the laminar cribrosa in the eyes of these small animals (27). The ability to access refined measurements of glaucoma pathology in a longitudinal and non-invasive manner could improve the usability of these models moving forward (91–93).

In addition, the recent development of novel imaging techniques has resulted in huge opportunities for data-driven AI approaches for basic science studies. For example, the recent advancements of adaptive optics, which is adopted from telescope technology, and two-photon imaging (94–96) have enabled *in vivo* visualization of glaucoma-related ocular structures such as the retina, ONH and trabecular meshwork in unprecedented detail (97). These data can facilitate efficient and accurate retinal layer segmentations (98, 99), cellular-level imaging of photoreceptors (100), and detect subtle pathological protein deposition in RNFL in both human (101) and animal studies (102). Furthermore, transmission electron microscopy and laser scanning microscopy can image the ultrastructural morphology of the ONH (103, 104), trabecular meshwork (105), and RNFL (106) from mouse and non-human primate models of glaucoma, enabling more in-depth understanding of the glaucoma pathogenesis (105) including astrocytic responses (104). While the large amounts of high-resolution imaging data pose analytic challenges using traditional image analysis methods, the fast-growing field of AI application in digital pathology offers a potential solution (107–109). Increased collection and digitalization of ophthalmic imaging from both human and experimental animal specimens provides great opportunities for harnessing reverse AI translation to evaluate glaucoma pathogenesis on a large scale with improved robustness.

Finally, the recent development of efficient sequencing techniques has enabled AI applications on multi-omics data for basic glaucoma research. For instance, genome-wide association studies (GWAS) have revealed hundreds of POAG-related genetic loci with consistent effects across ancestries (23, 110, 111); and whole-genome-based polygenic risk score enables the prediction of future glaucoma risks (112). Furthermore, multi-omics investigations can help identify the molecular signature for glaucoma predisposition (113), patient-specific tear composition (114), mechanical stress-derived trabecular meshwork cytoskeletal changes (115), and risk factors for IOP elevation (116). Integrating image data with multi-omics data (e.g., genomics, transcriptomics, proteomics, and metabolomics) using approaches such as spatial transcriptomics (117) may reveal novel genotype-phenotype associations and causal inferences, allowing the understanding of glaucoma-related disease etiology in a highly localized manner as well as the identification of more biologically-related glaucoma phenotypes.

## Data Availability

The original contributions presented in the study are included in the article/supplementary material. Further inquiries can be directed to the corresponding authors.
